# Supplementary material for: Transcriptomic and Proteomic Insights into the Effect of Sterigmatocystin on Aspergillus flavus
Source: J Fungi (Basel). 2023 Dec 13;9(12):1193. doi: 10.3390/jof9121193 (PMC10745003; doi:10.3390/jof9121193)
Supplement: Supplementary file 1 [file jof-09-01193-s001.zip › jof-2700474-supplementary.pdf]

Transcriptomic and Proteomic Analysis of *Aspergillus flavus* Response to Sterigmatocystin

Table S1. Proportions of genes with different expression levels.

Samples	FPKM Interval				
	0~1	1~3	3~15	15~60	＞60
CK_1	233	399	1041	833	872
CK_2	220	407	1032	818	853
CK_4	251	423	1048	829	788
TJ_1	697	489	722	637	614
TJ_2	754	463	728	655	587
TJ_4	594	473	753	673	684

Table S2. GO functional enrichment analysis of differentially expressed genes.

GO ID	Number	Pop number	P value_uncorrected	FDR	Description
			**MF**		
GO:0016491	548	1573	4.81297E-10	0	Oxidoreductase activity
GO:0003824	1480	5098	6.51185E-10	0	catalytic activity
GO:0016705	119	287	9.20882E-09	0	oxidoreductase activity, acting on paired donors, with incorporation or reduction of molecular oxygen
GO:0003735	6	113	1.43745E-08	0	structural constituent of ribosome
GO:0004497	119	291	1.88615E-08	0	monooxygenase activity
			**BP**		
GO:0071840	53	413	2.58209E-10	0	cellular component organization or biogenesis
GO:0055114	543	1538	4.10448E-10	0	oxidation-reduction process
GO:0044710	918	2926	5.87454E-10	0	single-organism metabolic process
GO:0044699	1217	4145	6.47599E-10	0	single-organism process
GO:0044267	76	504	1.37828E-09	0	cellular protein metabolic process
			**CC**		
GO:0044446	98	667	3.2679E-10	0	intracellular organelle part
GO:0044422	98	672	3.71546E-10	0	organelle part
GO:0032991	168	996	4.08468E-10	0	macromolecular complex
GO:0044424	456	2232	5.59664E-10	0	intracellular part
GO:0044464	538	2507	9.22963E-10	0	cell part

Note: FDR: P-value corrected.

Table S3. Top 10 KEGG enrichments for up and downregulated genes.

Ko id	Term	Number	P value_uncorrected
	**Downregulated genes**		
map00254	Aflatoxin biosynthesis	13	0.000
map00500	Starch and sucrose metabolism	20	0.001
map00350	Tyrosine metabolism	18	0.001
map00650	Butanoate metabolism	12	0.001
map00380	Tryptophan metabolism	15	0.002
map00071	Fatty acid degradation	12	0.005
map02010	ABC transporters	5	0.006
map04213	Longevity regulating pathway - multiple	8	0.007
map00591	Linoleic acid metabolism	3	0.011
map00061	Fatty acid biosynthesis	7	0.012
	**Upregulated genes**		
map00290	Valine, leucine and isoleucine	14	0.000
map00250	Alanine, aspartate and glutamate	16	0.000
map04113	Meiosis – yeast	18	0.000
map00770	Pantothenate and CoA biosynthesis	11	0.000
map00970	Aminoacyl-tRNA biosynthesis	15	0.000
map00670	One carbon pool by folate	8	0.000
map00300	Lysine biosynthesis	8	0.001
map03008	Ribosome biogenesis in eukaryotes	18	0.001
map00230	Purine metabolism	23	0.002
map00260	Glycine, serine, and threonine	18	0.003

Table S4. Protein Information for the six samples

Samples	Concentration (mg/mL)	total amount (mg)
TJ-3	5.588	0.950
TJ-5	6.207	1.055
TJ-6	5.313	0.903
CK-3	4.434	0.754
CK-5	6.750	1.148
CK-6	6.457	1.098

Table S5. Sixty-seven KEGG pathways associated with differentially expressed proteins

Pathway	Protein number	Background protein number	P value
**Metabolism**			
Aflatoxin biosynthesis	7	12	1.37E-05
Glycolysis / Gluconeogenesis	11	34	4.44E-05
Glutathione metabolism	9	24	6.29E-05
Carbon metabolism	18	84	7.60E-05
Glyoxylate and dicarboxylate metabolism	10	31	0.000103216
Tryptophan metabolism	7	19	0.000486853
Starch and sucrose metabolism	7	20	0.000688923
Methane metabolism	6	18	0.002213504
Fructose and mannose metabolism	6	21	0.005020163
Biosynthesis of antibiotics	24	186	0.008248332
Valine, leucine, and isoleucine degradation	6	25	0.011668305
Oxidative phosphorylation	1	64	0.018098524
Ascorbate and aldarate metabolism	2	3	0.019685684
Ether lipid metabolism	2	4	0.036084654
Fatty acid degradation	4	17	0.038473425
Pyruvate metabolism	6	35	0.045518011
Pantothenate and CoA biosynthesis	3	11	0.049321427
Histidine metabolism	3	11	0.049321427
Glycerophospholipid metabolism	4	19	0.05271297
Glycine, serine, and threonine metabolism	5	28	0.055717632
Inositol phosphate metabolism	3	12	0.060291444
Thiamine metabolism	3	12	0.060291444
Nitrogen metabolism	3	12	0.060291444
Pentose and glucuronate interconversions	3	13	0.071853089
beta-Alanine metabolism	3	14	0.083828604
Biosynthesis of amino acids	8	103	0.145793378

Pentose phosphate pathway	3	20	0.15549855
Glycosylphosphatidylinositol (GPI)-anchor biosynthesis	1	2	0.155708289
Glycerolipid metabolism	2	11	0.179287714
One carbon pool by folate	2	12	0.197057823
Phenylalanine metabolism	2	12	0.197057823
Valine, leucine, and isoleucine biosynthesis	2	13	0.213290221
Arachidonic acid metabolism	1	3	0.21387675
alpha-Linolenic acid metabolism	1	3	0.21387675
Alanine, aspartate, and glutamate metabolism	1	27	0.226971283
Lysine degradation	2	14	0.227881441
Cysteine and methionine metabolism	3	34	0.237720437
Pyrimidine metabolism	2	33	0.244638617
2-Oxocarboxylic acid metabolism	2	33	0.244638617
Amino sugar and nucleotide sugar metabolism	1	25	0.25160071
Tyrosine metabolism	2	16	0.251956094
Biotin metabolism	1	4	0.261112564
Arginine and proline metabolism	2	18	0.269258125
Citrate cycle (TCA cycle)	1	22	0.28984601
Ubiquinone and other terpenoid-quinone biosynthesis	1	5	0.298832651
Cyanoamino acid metabolism	1	5	0.298832651
Fatty acid metabolism	1	20	0.315191469
Vitamin B6 metabolism	1	6	0.328295025
Arginine biosynthesis	1	17	0.350284875
Selenocompound metabolism	1	8	0.366781163
Porphyrin and chlorophyll metabolism	1	14	0.376880844
Butanoate metabolism	1	14	0.376880844
Terpenoid backbone	1	14	0.376880844

biosynthesis			
Biosynthesis of unsaturated fatty acids	1	9	0.377666438
Sulfur metabolism	1	13	0.382515276
Riboflavin metabolism	1	10	0.384042432
**Cellular Processes**			
Peroxisome	7	30	0.007807138
Autophagy	1	33	0.161340442
Phagosome	1	27	0.226971283
**Genetic Information Processing**			
Spliceosome	1	48	0.059723004
Protein processing in endoplasmic reticulum	3	50	0.18634104
Aminoacyl-tRNA biosynthesis	1	28	0.215093738
RNA degradation	3	28	0.221251643
Ribosome biogenesis in eukaryotes	1	26	0.239155944
Sulfur relay system	1	4	0.261112564
Ubiquitin mediated proteolysis	1	17	0.350284875
**Environmental Information Processing**			
MAPK signalling pathway	3	28	0.221251643
